# Factors Contributing to the Severity and Laterality of Pisa Syndrome in Parkinson’s Disease

**DOI:** 10.3389/fnagi.2021.716990

**Published:** 2022-01-03

**Authors:** Young Eun Huh, Dae-Won Seo, Kunhyun Kim, Won-Ho Chung, Seonwoo Kim, Jin Whan Cho

**Affiliations:** ^1^Department of Neurology, CHA Bundang Medical Center, CHA University, Seongnam, South Korea; ^2^Department of Neurology, Samsung Medical Center, Sungkyunkwan University School of Medicine, Seoul, South Korea; ^3^Department of Otolaryngology, Head and Neck Surgery, Samsung Medical Center, Sungkyunkwan University School of Medicine, Seoul, South Korea; ^4^Statistics and Data Center, Samsung Medical Center, Research Institute for Future Medicine, Seoul, South Korea

**Keywords:** Parkinson’s disease, pisa syndrome, misperception of verticality, asymmetry of motor symptoms, peripheral vestibular hypofunction

## Abstract

**Objective:** Pisa syndrome (PS) is a disabling postural deformity in Parkinson’s disease (PD). We aimed to elucidate clinical factors determining the severity and laterality of PS in PD.

**Methods:** In 54 PD patients with PS, we measured the clinical factors that are previously known to contribute to the occurrence of PS as follows: asymmetry of motor symptoms for the evaluation of asymmetric basal ganglia dysfunction, the degree and direction of subjective visual vertical (SVV) tilt for the misperception of body verticality, the canal paresis for unilateral peripheral vestibulopathy, and the tonic electromyographic (EMG) hyperactivity of paraspinal muscles for dystonia. Multivariable linear and logistic regression analyses were conducted to identify the clinical factors associated with the degree of truncal tilt, for the quantification of the severity of PS, and PS tilting to the less affected side, respectively.

**Results:** The multivariable linear regression analyses revealed that the larger degree of SVV tilt (β = 0.29, SE = 0.10, *p* = 0.005), right-sided SVV tilt (β = 2.32, SE = 0.82, *p* = 0.007), and higher Hoehn and Yahr (HY) stage (β = 4.01, SE = 1.29, *p* = 0.003) significantly increased the severity of PS. In the multivariable logistic regression analyses, greater asymmetry of motor symptoms [odds ratio (OR) = 2.01, 95% CI = 1.34–3.49] was significantly associated with PS tilting to the less affected side, while right-sided SVV tilt (OR = 0.02, 95% CI = 0.001–0.21), unilateral canal paresis (OR = 0.06, 95% CI = 0.003–0.79), and higher HY stage (OR = 0.04, 95% CI = 0.002–0.46) were associated with PS tilting to the more affected side.

**Conclusion:** Misperception of verticality, asymmetric basal ganglia dysfunction, unilateral peripheral vestibulopathy, and motor disability are the clinical factors associated with the severity and laterality of PS in patients with PD.

## Introduction

Pisa syndrome (PS) is defined as a reversible lateral flexion of the trunk that can be diminished by passive movement or supine positioning (Castrioto et al., 2014; Barone et al., 2016). It is a devastating postural deformity in patients with Parkinson’s disease (PD) as it causes dyspnea, postural imbalance, and falls.

Pathomechanisms underlying PS in patients with PD are considered to be multifactorial. Several hypotheses have been suggested to explain the occurrence of PS based on both clinical and experimental data. For instance, asymmetric basal ganglia dysfunction is classically believed to have a primary role in the pathogenesis of PS (Castrioto et al., 2014; Barone et al., 2016). This was because PD patients with PS seemed to have greater asymmetry of motor symptoms or seemed to lean toward the side of the body with less severe motor symptoms (Di Matteo et al., 2011; Vitale et al., 2011; Tassorelli et al., 2012). Recent studies suggested that the failures of sensorimotor integration and disrupted spatial orientation are central players in the generation of PS (Castrioto et al., 2014; Scocco et al., 2014; Tinazzi et al., 2015; Barone et al., 2016; Gandor et al., 2016; Huh et al., 2018). Our previous study also showed that the misperception of the orientation on verticality was strongly associated with the presence of PS in patients with PD (Huh et al., 2018). Unilateral peripheral vestibular hypofunction has also been reported to cause PS in patients with PD, leading exclusively to lateral truncal flexion toward the same side as the peripheral vestibular loss (Mamo et al., 1965; Vitale et al., 2011). Several studies have suggested dystonia as an etiology of PS based on the tonic electromyographic (EMG) activity observed in the paraspinal or abdominal muscles on the same side as the direction of truncal tilt (Di Matteo et al., 2011; Tassorelli et al., 2012; Tinazzi et al., 2013). Higher cortical dysfunction, such as attention deficits and visuospatial impairment, has also been associated with the occurrence of PS in patients with PD (Vitale et al., 2016; Artusi et al., 2019).

In contrast to intensive investigation on the occurrence of PS, less is known about the factors associated with specific aspects of PS, such as its severity and laterality (i.e., the direction of truncal tilt). Elucidating the main determinants of the severity and laterality of PS will increase our understanding on the pathogenesis of PS in PD. Moreover, factors associated with the severity of PS may help early detection of PS at a higher risk for more severe PS because PS mostly develops chronically, with subtle initial postural deformity and gradual progression.

In this study, we aimed to evaluate the clinical factors associated with the severity of PS in patients with PD. We also sought to determine factors related to the laterality of PS, such as those associated with PS tilting to the less affected side and those associated with PS tilting to the right. In this study, we measured potential risk factors that are previously known to contribute to the occurrence of PS, such as asymmetric basal ganglia dysfunction, misperception of verticality, unilateral peripheral vestibular hypofunction, and dystonia. Then, we validated whether these factors were associated with the severity and laterality of PS. We also tested other clinical variables of potential relevance to PS, such as motor disability, for their association with the severity and laterality of PS.

## Materials and Methods

### Participants

We included 54 PD patients with PS at the Movement Disorders Clinic of Samsung Medical Center from January 2016 to October 2017. This cohort was composed of all 54 patients with PD-PS from our previous work, and the detailed characteristics of the entire patients with PD have been described elsewhere (Huh et al., 2018). All patients with PD fulfilled the UK Parkinson’s Disease Society Brain Bank criteria for idiopathic PD (Hughes et al., 1992). The exclusion criteria were as follows: (1) postural deformities other than PS, such as camptocormia (≥ 45°), anterocollis (≥ 45°), and retrocollis (Doherty et al., 2011); (2) severe dyskinesia; (3) exposure to medications potentially causing PS, such as antiemetics, neuroleptics, antidepressants, and central cholinergic inhibitors; (4) ocular misalignment or visual loss; (5) history (i.e., acute vertigo with nausea/vomiting) or clinical signs (i.e., spontaneous nystagmus with/without visual fixation), suggesting acute vestibular disorders (e.g., acute vestibular neuronitis); (6) signs of somatosensory disturbances, such as reduced response of deep tendon reflex; (7) major orthopedic problems disclosed by spine X-ray; (8) dementia; (9) neurosurgical intervention; and (10) comorbid neurological disorders possibly affecting posture.

PD patients with PS (PD-PS patients) were defined as patients with PD with a lateral tilt of the trunk ≥ 10° that could be alleviated by supine positioning or by passive mobilization (Castrioto et al., 2014; Barone et al., 2016). The degree of lateral truncal tilt was determined by measuring the angle between a vertical line on the wall and an assumptive line passing through the C7 and L4 points in the back while the patient was standing (Huh et al., 2018). Truncal tilt was recorded by using a digital camera placed 2 m behind the patient at a height of 1 m. PD-PS patients who tilted toward the side of the body with less severe motor symptoms were classified as PD-PS patients tilting to the less affected side and vice versa.

We used the Unified Parkinson’s Disease Rating Scale motor score (UPDRS-III) to evaluate the motor disability. The asymmetry score of motor symptoms was assessed by calculating the summed difference of the score between the right and left item 20–26 from UPDRS-III at the time of examination (Tinazzi et al., 2014, 2015). The dominant side of motor symptoms at the disease onset was documented. Clinical postural balance was evaluated by using Berg Balance Scale (BBS), a 56-point scale in which lower scores indicate poorer balance (Johnson et al., 2013). We tested global cognitive functions using the Mini-Mental State Examination (MMSE). We estimated levodopa equivalent daily dose (LEDD) and categorized treatment regimens based on the use of levodopa or dopamine agonists (Tomlinson et al., 2010). The presence of back pain was identified when it lasted > 12 months, and its severity was ≥ 5 on a visual analog scale. All evaluations were conducted during the on-medication state.

### Subjective Visual Vertical Testing

Perception of body verticality was assessed by using the subjective visual vertical (SVV) tests (Yang et al., 2014). SVV testing, which primarily relies on the gravitational signals from vestibular otolithic and somatosensory systems, is widely used to measure the perceived body verticality (Bronstein, 1999). The detailed protocol was described previously (Huh et al., 2018). In brief, patients sat in a dark room in front of a computer screen positioned 1 m from their eyes. A rod was randomly displayed on the screen at various angles deviating from vertical. Patients viewed the rod (8.4-cm long and 0.4-cm wide) through a hole (15 cm in diameter) in a black panel installed before the screen to remove visual inference of verticality from the surroundings. They were instructed to sit upright while keeping their head upright. The examiner, who was blinded to the position of the rod, used a computer mouse to rotate the rod following the verbal instruction of patients to move the rod into an exact vertical position. The average of off-vertical angles from 10 trials was used to determine SVV tilt (in degrees). SVV tilt was considered normal when its degree was within the normal range (less than 3.0° to the right or left) obtained from 40 healthy control (20 men; mean age: 57.2 years; range: 45–72).

### Electromyographic Analysis

Multichannel EMG recordings were performed by using a conventional EMG machine (Viking IV, Nicolet Biomedicals, Madison, WI, United States). We recorded EMG activity from bilateral paraspinal thoracolumbar (T12-L1) muscles using silver-silver chloride surface electrodes while patients stood in a relaxed posture (Di Matteo et al., 2011). Amplified EMG signals were bandpass-filtered between 100 and 2,000 Hz. EMG recordings were sampled for 60 s in 15 consecutive 4-s sweeps. The presence of tonic EMG discharges was defined as EMG hyperactivity, indicating continuous muscle contraction.

### Bithermal Caloric Tests

For caloric stimulations, each ear was irrigated with cold (30.5°C) and warm (43.5°C) air. During the stimulation, the patients were lying in the supine position with their head elevated 30° so that both the horizontal semicircular canals were positioned in the vertical plane. During each stimulation, nystagmus was gradually built up, reached at its peak slow-phase velocity, and then gradually subsided. The peak slow-phase velocity of each stimulation on each ear is used as a vestibular response for each condition, such as vestibular response for warm stimulation on the right ear (RW), cold stimulation on the right ear (RC), warm stimulation on the left ear (LW), and cold stimulation on the left ear (LC). Jongkee’s formula was used to calculate the asymmetries of vestibular response between the ears as follows: 100 * ((LC + LW)-(RC + RW))/(RC + LC + RW + LW). The asymmetries of vestibular response > 25% were considered canal paresis (Moon et al., 2012).

### Statistical Analysis

For this study, the linear regression analyses were used for the continuous dependent variable (i.e., the degree of truncal tilt) while the logistic regression analyses were used for the categorical dependent variable (i.e., the laterality of truncal tilt). To assess the clinical factors associated with the severity of PS, we conducted univariable and multivariable linear regression analyses for the dependent variable of the degree of truncal tilt. The primary predictors were the asymmetry score of motor symptoms, the dominant side of motor symptoms, the degree of SVV tilt, the direction of SVV tilt, canal paresis, and the EMG hyperactivity of paraspinal muscles. Residuals for the degree of truncal tilt from values predicted by the model were examined to check the assumptions of normality and homoscedasticity. We also assessed the clinical factors related to the laterality of PS using univariable and multivariable logistic regression analyses. For this, the dependent variable was set as PS titling to the less affected side or PS titling to the right, as the larger number of participants were included in these groups (i.e., 59.3% of PD-PS patients tilted to the less affected side and 57.4% tilted to the right). The regression analysis was performed for PS tilting to the right to examine its association with the clinical factors with the laterality, such as right- and left-sided SVV tilt and right- and left-sided dominance of motor symptoms. The primary predictors were the same as described earlier. The reference level was set for the categorical independent variables, such as sex (male vs. female), PD motor subtype (postural instability gait disorder vs. tremor dominant), the dominant side of motor symptoms (right vs. left), the direction of SVV tilt (right-sided vs. normal vs. left-sided SVV tilt), canal paresis (unilateral vs. absence), EMG hyperactivity of paraspinal muscles (unilateral vs. bilateral), back pain (presence vs. absence), and treatment regimen (dopamine agonist vs. levodopa plus dopamine agonist vs. levodopa). The latter or the last value was set as the reference level for each variable. Multivariable analyses included all primary predictors and variables with *p* < 0.05 from the univariable analysis. To exclude variables with a high level of multicollinearity, variance influence factors (VIFs) were checked, and all variables included in multivariable models showed VIF < 4. However, as PD-PS patients whose degree of SVV tilt was less than 3 were also classified as those with normal SVV tilt, primary predictors of the degree of SVV tilt and the direction of SVV tilt were entered separately into multivariable models. For all tests, *p* < 0.05 was considered statistically significant. Bonferroni’s correction was used for the correction of multiple comparisons. Statistical analysis was performed using R (version 3.5.2).

## Results

The demographics and clinical characteristics of 54 PD-PS patients are summarized in Table 1. The degree of truncal tilt in PD-PS patients was 15.1 ± 3.1 (mean ± SD), ranging from 12 to 22. Of note, 32 (59.3%) PD-PS patients leaned toward the less affected side, while 31 (57.4%) PD-PS patients tilted to the right. Among 32 PD-PS patients tilting to the less affected side, 18 tilted to the right. The degree of SVV tilt was 6.0 ± 3.9 and ranged from 0.4 to 17.5. Right-sided SVV tilt was observed in 22 PD-PS patients (40.7%) and left-sided SVV tilt was found in 23 PD-PS patients (42.6%), while 9 PD-PS patients showed normal SVV tilt. Eight (14.8%) PD-PS patients showed unilateral canal paresis, all in the same direction as the truncal tilt of PS, consistent with the previous study (Vitale et al., 2011). All PD-PS patients showed either unilateral (*n* = 38, 70.4%) or bilateral (*n* = 16, 29.6%) EMG hyperactivity of paraspinal muscles. All 38 PD-PS patients with unilateral EMG hyperactivity exclusively exhibited EMG hyperactivity in the opposite direction of truncal tilt of PS.

**TABLE 1:** Univariable analysis of clinical factors associated with the severity of Pisa syndrome (PS) in patients with Parkinson’s disease (PD).

	PD-PS (*n* = 54)	β-coefficient	Standard error	*p*
**Age, years**	66.4 ± 6.4	0.14	0.06	0.037
**Male, n (%)**	33 (61.1)	0.24	0.86	0.780
**Disease duration, years**	7.9 ± 3.4	0.10	0.12	0.409
**Hoehn and Yahr stage**	2.2 ± 0.3	4.96	1.09	<0.001
**UPDRS-III**	22.8 ± 5.8	0.20	0.07	0.004
**PD motor subtype, PIGD, n (%)**	23 (42.6)	−0.56	0.85	0.512
**Asymmetry score of motor symptoms**	5.5 ± 2.6	0.16	0.16	0.333
**Dominant side of motor symptoms, right, n (%)**	32 (59.3)	0.79	0.85	0.359
**Degree of SVV tilt**	6.0 ± 3.9	0.38	0.10	<0.001
**Direction of SVV tilt, n (%)**				
Right-sided SVV tilt	22 (40.7)	3.04	0.82	0.002
Normal SVV tilt	9 (16.7)	0.51	1.08	>0.999
Left-sided SVV tilt*	23 (42.6)	NA	NA	NA
**Unilateral canal paresis, n (%)**	8 (14.8)	−0.03	1.19	0.982
**Unilateral EMG hyperactivity of paraspinal muscles, n (%)**	38 (70.4)	1.99	0.88	0.028
**Back pain, n (%)**	25 (46.3)	0.69	0.84	0.413
**BBS**	49.4 ± 3.1	−0.31	0.13	0.022
**LEDD, mg**	593.7 ± 218.0	0.001	0.002	0.916
**Treatment regimen, n (%)**				
Dopamine agonist	6 (11.1)	−2.75	1.96	0.332
Levodopa + dopamine agonist	44 (81.5)	−2.82	1.59	0.162
Levodopa*	4 (7.4)	NA	NA	NA
**BMI, kg/m^2^**	24.1 ± 2.6	−0.001	0.16	0.993
**MMSE**	28.0 ± 1.9	−0.18	0.23	0.435
**Education, years**	11.6 ± 3.6	0.15	0.12	0.200

*BBS, Berg Balance Scale; BMI, body mass index; LEDD, levodopa equivalent daily dose; MMSE, Mini-Mental State Examination; PIGD, postural instability and gait disturbance; SVV, subjective visual vertical; UPDRS-III, Unified Parkinson’s Disease Rating Scale motor score. Data are given in mean ± SD, unless otherwise noted.*

*Reference.

### Clinical Factors Associated With the Severity of Pisa Syndrome

**FIGURE 1:**
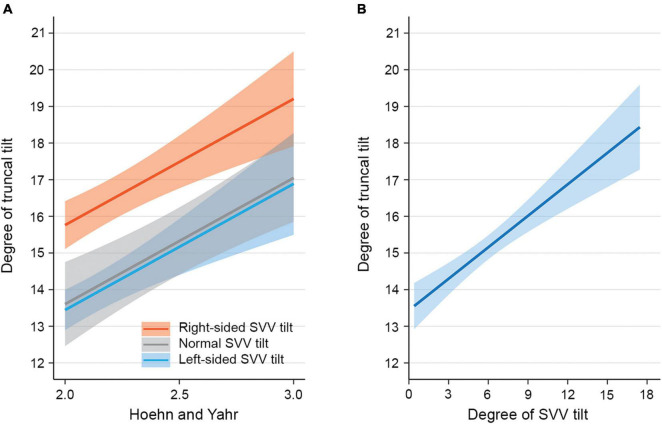
Model-predicted degree of truncal tilt in patients with Parkinson’s disease (PD) and Pisa syndrome (PS). **(A)** The estimated mean degree of truncal tilt (solid lines) with SEs (shaded area) from a multivariable linear regression model including the direction of subjective visual vertical (SVV) tilt. The estimated mean degree of truncal tilt is illustrated over Hoehn and Yahr stage for each PD-PS patient subgroup with right-sided SVV tilt, normal SVV tilt, and left-sided SVV tilt. The estimates are adjusted for covariates such as age, Berg Balance Scale (BBS), Unified Parkinson Disease Rating Scale motor score (UPDRS-III), asymmetry score of motor symptoms, right-sided dominance of motor symptoms, unilateral canal paresis, and unilateral electromyographic (EMG) hyperactivity of paraspinal muscles. **(B)** The estimated mean degree of truncal tilt (solid line) with SEs (shaded area) from a multivariable linear regression model including the degree of SVV tilt. The estimated mean degree of truncal tilt is illustrated over the degree of SVV tilt. The estimates are adjusted for covariates such as age, BBS, UPDRS-III, asymmetry score of motor symptoms, right-sided dominance of motor symptoms, unilateral canal paresis, and unilateral EMG hyperactivity of paraspinal muscles.

In the univariable linear regression analysis for factors associated with the severity of PS, the larger degree of SVV tilt, right-sided SVV tilt, contralateral EMG hyperactivity of paraspinal muscles, older age, higher HY stage, higher UPDRS-III scores, and lower BBS were significantly associated with the larger degree of truncal tilt in PD-PS patients (Table 1). For the multivariable linear regression analyses, primary predictors described earlier and covariates of age, HY stage, UPDRS-III, and BBS were used as independent variables. A multivariable linear regression model including the direction of SVV tilt revealed that right-sided SVV tilt (β = 2.32, SE = 0.82, *p* = 0.007) and higher HY stage (β = 3.45, SE = 1.46, *p* = 0.023) significantly increased the degree of truncal tilt in PD-PS patients (Figure 1A). Specifically, PD-PS patients with right-sided SVV tilt showed a 2.32 higher degree of truncal tilt compared to those with left-sided SVV tilt, and the degree of truncal tilt was increased by 3.45 degree with an increment of one HY stage. A multivariable linear regression model including the degree of SVV tilt showed a strong positive linear association between the degree of SVV tilt and the degree of truncal tilt in PD-PS patients, by increasing 0.29 degree of truncal tilt for an increase in one degree of SVV tilt (β = 0.29, SE = 0.10, *p* = 0.005) (Figure 1B). HY stage was also positively associated with degree of truncal tilt in this model (β = 4.01, SE = 1.29, *p* = 0.003).

### Clinical Factors Associated With Pisa Syndrome Tilting to the Less Affected Side

**FIGURE 2:**
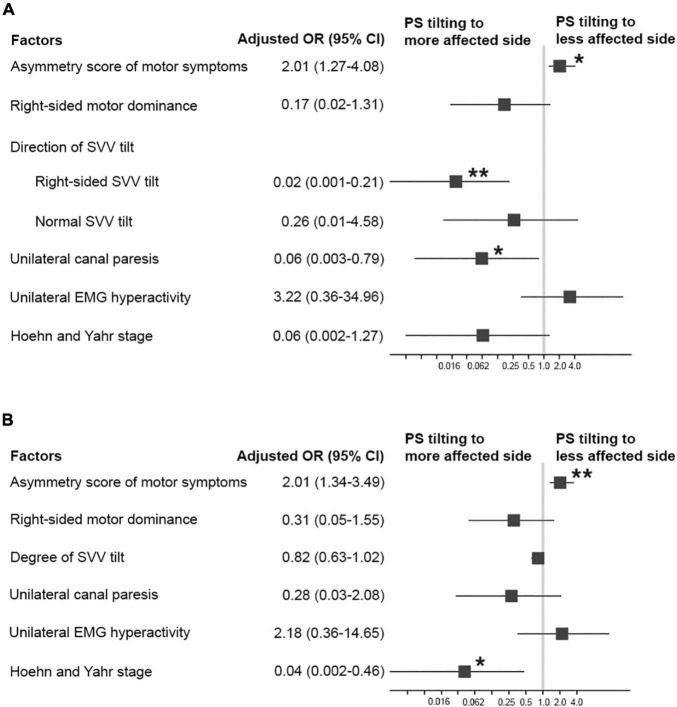
Factors associated with PS tilting to the less affected side in patients with PD. **(A)** Adjusted odds ratios (ORs; square) and 95% CI (error bars) from a multivariable logistic regression model including the *direction* of SVV tilt. **(B)** Adjusted ORs (square) and 95% CI (error bars) from a multivariable logistic regression model including the *degree* of SVV tilt. *p < 0.05, **p < 0.01.

In the univariable logistic regression analysis for factors associated with PS tiling to the less affected side, a higher asymmetry score of motor symptoms was significantly associated with the PS tilting to the less affected side. On the contrary, right-sided dominance of motor symptoms, unilateral canal paresis, and higher HY stage were significantly associated with the PS tilting to the more affected side (Table 2). The multivariable logistic regression analyses were conducted by using primary predictors and a covariate of the HY stage as independent variables. In a multivariable logistic regression model including the direction of SVV tilt, a higher asymmetry score of motor symptoms was significantly associated with PS tilting to the less affected side [odds ratio (OR) = 2.01, 95% CI 1.27–4.08, *p* = 0.013] (Figure 2A). On the contrary, right-sided SVV tilt (OR = 0.02, 95% CI 0.001–0.21, *p* = 0.006) and unilateral canal paresis (OR = 0.06, 95% CI 0.003–0.79, *p* = 0.043) were significantly associated with PS tilting to the more affected side (Figure 2A). A multivariable model run with the degree of SVV tilt demonstrated that the higher asymmetry score of motor symptoms was significantly related to PS tilting to the less affected side (OR = 2.01, 95% CI 1.34–3.49, *p* = 0.003) while higher HY stage was significantly related to PS tilting to the more affected side (OR = 0.04, 95% CI 0.002–0.46, *p* = 0.019) (Figure 2B).

**TABLE 2:** Univariable analysis of clinical factors associated with PS tilting to the less affected side.

	PS tilting to the less affected side (*n* = 32)	PS tilting to the more affected side (*n* = 22)	OR	95% CI for OR	*p*
**Age, years**	65.8 ± 7.1	67.4 ± 5.4	0.96	0.88–1.05	0.383
**Male gender, n (%)**	19 (59.4)	14 (63.6)	0.84	0.27–2.54	0.752
**Disease duration, years**	7.5 ± 3.4	8.6 ± 3.4	0.91	0.77–1.07	0.247
**Hoehn and Yahr stage**	2.1 ± 0.3	2.3 ± 0.4	0.08	0.01–0.47	0.010
**UPDRS-III**	21.8 ± 5.2	24.4 ± 6.4	0.92	0.82–1.02	0.120
**PD motor subtype, PIGD, n (%)**	13 (40.6)	10 (45.5)	0.82	0.27–2.48	0.724
**Asymmetry score of motor symptoms**	6.5 ± 2.6	4.0 ± 2.0	1.63	1.19–2.24	0.002
**Dominant side of motor symptoms, right, n (%)**	15 (46.9)	17 (77.3)	0.26	0.07–0.83	0.030
**Degree of SVV tilt**	5.7 ± 3.6	6.4 ± 4.5	0.96	0.83–1.10	0.546
**Direction of SVV tilt, n (%)**					
Right-sided SVV tilt	9 (28.1)	13 (59.1)	0.24	0.07–0.83	0.056
Normal SVV tilt	6 (18.8)	3 (13.6)	0.71	0.14–4.16	> 0.999
Left-sided SVV tilt*	17 (53.1)	6 (27.3)	NA	NA	NA
**Unilateral canal paresis, n (%)**	2 (6.3)	6 (27.3)	0.18	0.02–0.87	0.048
**Unilateral EMG hyperactivity of paraspinal muscles, n (%)**	23 (71.9)	15 (68.2)	1.19	0.36–3.90	0.770
**Back pain, n (%)**	14 (43.8)	11 (50)	0.78	0.26–2.32	0.651
**BBS**	49.8 ± 2.7	48.8 ± 3.4	1.12	0.93–1.35	0.239
**LEDD, mg**	563.4 ± 225.6	637.7 ± 203.4	0.10	0.10–1.00	0.221
**Treatment regimen, n (%)**					
Dopamine agonist	2 (6.3)	2 (9.1)	2.00	0.14–31.73	> 0.999
Levodopa + dopamine agonist	26 (81.3)	18 (81.8)	1.44	0.16–12.96	> 0.999
Levodopa*	4 (12.5)	2 (9.1)	NA	NA	NA
**BMI, kg/m^2^**	24.4 ± 2.6	23.8 ± 2.8	1.08	0.88–1.34	0.458
**MMSE**	28.0 ± 1.9	27.9 ± 1.8	1.05	0.78–1.42	0.742
**Education, years**	11.0 ± 3.3	12.4 ± 4.0	0.90	0.76–1.05	0.170

*BBS, Berg Balance Scale; BMI, body mass index; LEDD, levodopa equivalent daily dose; MMSE, Mini-Mental State Examination; PIGD, postural instability and gait disturbance; SVV, subjective visual vertical; UPDRS-III, Unified Parkinson’s Disease Rating Scale motor score. Data are given in mean ± SD, unless otherwise noted.*

*Reference.

### Clinical Factors Associated With Pisa Syndrome Tilting to the Right

In the univariable logistic regression analysis for factors associated with PS tilting to the right, the right-sided SVV tilt was significantly associated with PS tilting to the right while the right-sided dominance of motor symptoms was significantly associated with PS tilting to the left (Supplementary Table 1). Primary predictors were used as independent variables for the multivariable logistic regression analyses. In a multivariable logistic regression model including the direction of SVV tilt, PD-PS patients with right-sided SVV tilt approximately 2.5 times more frequently tilted to the right (OR = 12.91, 95% CI 2.77–81.10, *p* = 0.002). A multivariable model including the degree of SVV tilt demonstrated that the right-sided dominance of motor symptoms was significantly associated with PS tilting to the left (OR = 0.27, 95% CI 0.07–0.90, *p* = 0.042).

## Discussion

We found that the larger degree of SVV tilt, right-sided SVV tilt, and more severe motor disability significantly increased the severity of PS in patients with PD. Greater asymmetry of motor symptom was significantly associated with PS tilting to the less affected side, while right-sided SVV tilt, unilateral canal paresis, and more severe motor disability were significantly associated with PS tilting to the more affected side. In addition, PD-PS patients with right-sided SVV tilt more frequently tilted to the right. Overall, this study showed that the potential risk factors for the occurrence of PS, such as misperception on verticality, asymmetric basal ganglia dysfunction, and peripheral vestibular hypofunction, are also significantly associated with the severity and laterality of PS in patients with PD.

To the best of our knowledge, this is the first study to validate whether potential risk factors for the occurrence of PS may contribute to the specific features of PS in patients with PD. This study provided strong corroborative evidence for the present hypothesis on the pathogenesis of PS, such as asymmetric basal ganglia dysfunction, misperception on body verticality, and unilateral peripheral vestibular hypofunction. Of importance, these data shed some light on the detection of patients with PD at a higher risk for more severe PS, such as misperception on verticality and motor disability.

In this study, misperception on verticality was significantly associated with both the severity and laterality of PS in patients with PD. Normal perception on verticality is essential for postural control that requires accurate integration of sensory inputs from the visual, vestibular, and somatosensory systems (Horak, 2006). Recent studies suggested that the misperception on verticality is strongly related to the presence of PS in patients with PD (Scocco et al., 2014; Tinazzi et al., 2015; Gandor et al., 2016; Brugger et al., 2020). For example, the veering during the straight-walking test with eyes closed was significantly associated with PS in patients with PD (Tinazzi et al., 2015). In our previous study, abnormal deviation of SVV was significantly more frequent in PD patients with PS compared to those without PS (Huh et al., 2018). Similarly, PD patients with PS showed a larger degree of SVV tilt compared to those without PS (Scocco et al., 2014; Brugger et al., 2020). In addition to the presence of PS, this study revealed a significant influence of verticality misperception on the severity and laterality of PS. Together, the present result reinforced the pathogenic role of verticality misperception in the generation of PS in patients with PD.

This study showed that the severity of PS was significantly increased with the larger degree of SVV tilt, right-sided SVV tilt, and higher HY stage. A positive relationship between motor disability, or disease stage, and the severity of PS has been described in previous reports (Kashihara and Imamura, 2012; Geroin et al., 2015; Tinazzi et al., 2015). However, the association between the severity of PS and either the degree or the direction of SVV tilt has rarely been addressed. In this study, the greater degree of SVV tilt and right-sided SVV tilt were significantly associated with the more severe truncal tilt in PD-PS patients. As none of the PD-PS patients in our study showed any clinical signs of peripheral sensory deficits, abnormal SVV tilt in our PD-PS patients may be largely driven by damage to the central graviceptive pathway, consistent with the previous reports (Barnett-Cowan et al., 2010; Pereira et al., 2014; Brugger et al., 2020). Central graviceptive pathway starts from the vestibular nuclei, runs through the brainstem, and reaches out to higher subcortical and cortical regions, such as posterior lateral thalamus, basal ganglia, insular cortex, inferior frontal gyrus, superior temporal gyrus, and inferior parietal lobule (Baier et al., 2012; Yang et al., 2014). Unilateral damage to any of these regions can cause abnormal deviation of perceived verticality and can result in lateral body tilt through adaptive postural alignment in the same direction as the erroneous perception of verticality (Dieterich and Brandt, 1992; Perennou et al., 2008). Similarly, the asymmetric pathology of PD can induce abnormal tilt of perceived verticality and can lead to lateral truncal tilt in PD-PS patients, as observed in previous studies (Scocco et al., 2014; Huh et al., 2018; Brugger et al., 2020). Recently, Brugger et al. (2020) reported that asymmetrically reduced gray matter volume in the right thalamus is associated with a larger degree of SVV tilt in PD patients with PS. Assumably, a larger tilt of perceived verticality, due to the more asymmetric degeneration in graviceptive network, can lead to a more severe deviation of the vertical body axis in PD patients with PS. The right-sided SVV tilt may contribute to the more severe body tilt of PS probably because the degree of SVV tilt was greater in our PD-PS patients with the right-sided SVV tilt compared to those with left-sided SVV tilt (7.9° ± 4.2 vs. 5.6° ± 2.9, *p* = 0.016; Supplementary Table 2).

However, it is still unclear that how the severity of PS is associated with misperception on verticality. This is because changes in perceived verticality caused by chronic progressive hemispheric lesions may be rather variable compared to those induced by acute damage to the brainstem or peripheral otolithic organ (Perennou et al., 2008). In addition, the possible reciprocal interaction between the verticality misperception and the chronic postural misalignment should also be considered to interpret the present finding. Regardless of the specific pathomechanism involved, this result suggests that the testing for SVV may give a clue on the clinical biomarker that can help the early recognition of patients with PD at a higher risk for severe PS.

Asymmetric basal ganglia dysfunction has long been considered a primary pathomechanism of PS. This hypothesis mostly stems from previous observations on PD patients with PS who presented with more severe asymmetry of motor symptoms or leaned preferentially toward the less affected side (Di Matteo et al., 2011; Vitale et al., 2011; Tassorelli et al., 2012). However, a recent large-scale controlled study has reported neither laterality nor asymmetry of motor symptoms was associated with PS (Tinazzi et al., 2015). A recent autopsy study also failed to confirm the significant asymmetry of PD pathology in a PD patients with PS (Solla et al., 2016). In line with the previous findings, our previous study demonstrated that the asymmetry of motor symptoms did not differ significantly between PD patients with and without PS, and only about 60% of PD patients with PS leaned toward the less affected side (Huh et al., 2018). Even though a link between asymmetric basal ganglia dysfunction and the occurrence of PS is lacking, this study clearly showed a significant association between the greater asymmetry of motor symptoms and the preferential laterality of PS in the direction of the less affected side. Of interest, PD-PS patients tilting to the more affected side were significantly associated with risk factors for PS other than asymmetric basal ganglia dysfunction, such as misperception of verticality and unilateral peripheral vestibular hypofunction. In addition, the right-sided dominance of motor symptoms was associated with PS tiling to the left. This can be, in part, interpreted based on the clinical observations that patients with PD tended to tilt to the less affected side. However, this speculation is limited because the direct association between the right-sided dominance of motor symptoms and PS tilting to the less affected side was not identified in this study. Alternatively, these all indicate the heterogeneity of pathomechanism determining the laterality of PS in patients with PD. Collectively, the current finding, for the first time, provided a pathomechanistic link between asymmetric basal ganglia dysfunction and PS in patients with PD.

Vestibular hypofunction has been also reported to have a role in the pathogenesis of PS in patients with PD. In PD patients with PS, vestibulospinal function, measured by cervical vestibular evoked myogenic potentials, was more severely deteriorated compared to those without PS (Di Lazzaro et al., 2018). Previous studies evaluating peripheral vestibular function by using bithermal caloric test demonstrated that patients with PD-PS showed peripheral vestibular hypofunction more frequently compared to those without PS, presenting either unilateral or bilateral canal paresis (Vitale et al., 2011; Tang et al., 2021). In this study, even though the small number of PD-PS patients showed unilateral canal paresis, the unilateral peripheral vestibular hypofunction significantly contributed to the laterality of PS, especially PS tilting to the more affected side. This result supports the pathogenic role of peripheral vestibular hypofunction in the generation of PS and reinforces the heterogeneity of pathomechanism of PS in patients with PD.

In the previous studies, EMG hyperactivity of paraspinal or abdominal muscles has been observed either in the same side of the truncal tilt of PS or in its opposite side (Di Matteo et al., 2011; Tassorelli et al., 2012; Tinazzi et al., 2013). These EMG patterns have been interpreted as indicative of the dystonia etiology for PS or the compensatory muscle contraction, respectively. Until recently, EMG patterns have never been associated with the severity or laterality of PS in patients with PD. In this study, the patterns of paraspinal EMG hyperactivity were not associated with either the severity or laterality of PS. Of note, our PD-PS patients showed EMG patterns indicating the compensatory muscle contraction, rather than those supporting the dystonia hypothesis. These conflicting results might be due to methodological variability or different patient characteristics, such as ethnic difference. Even though the prevalence of PS in patients with PD needs further elucidation, recent studies showed that PS appeared less frequent in the Asian ancestry (3.6% in China) compared to in the European one (8.8% in Italy) (Tinazzi et al., 2015; Liu et al., 2019). The ethnic difference, involving the different genetic background or the unique lifestyle, can lead to the difference in the prevalence as well as the pathomechanisms of PS. For instance, *GBA* mutations that are associated with the more severe PD phenotype are more common in the European/North American ancestry compared to the Asian one (Tan et al., 2007; De Marco et al., 2008; Choi et al., 2012; Liu et al., 2016; Blandini et al., 2019). Assumably, specific genetic architecture may predispose patients with PD to the development of postural deformities, leading to a higher frequency of PS and more diverse etiologies, such as dystonia. To advance our understanding on the pathomechanism of PS, a multicenter study including multiple ethnicities is needed based on the unified methodological platform.

This study has several limitations. First, the number of PD patients with PS was relatively small to secure the statistical power. This is understandable as the frequency of PS among patients with PD has been reported to be only about 3–9% (Tinazzi et al., 2015; Liu et al., 2019). Nevertheless, we were able to obtain adequate statistical significance for clinical factors of interest after adjusting for other covariates. Future validation study using a larger patient population may help increase the statistical power of the present results. Second, even though the global cognitive function was assessed by using the MMSE in this study, the contribution of higher cognitive function, such as attention and visuospatial performance, was not evaluated (Vitale et al., 2016; Artusi et al., 2019). Third, the contribution of the peripheral vestibular hypofunction might be underestimated in the current study. We excluded the patients with the history or signs of acute peripheral vestibular disorders because the active peripheral vestibulopathy influences the verticality misperception and the postural alignment regardless of the presence of chronically progressing pathology (Vibert et al., 1999; Kim et al., 2008). Nevertheless, there is a possibility that unilateral vestibular hypofunction related to PS might be masked by that related to acute vestibular events and might be eliminated from this study. Finally, the cross-sectional design of this study did not allow the clarification on causality between risk factors and the specific features of PS. Based on the current promising cross-sectional results, a longitudinal study is needed to determine whether these clinical factors can virtually predict the severity or the laterality of PS.

## Conclusion

The misperception of verticality, asymmetric basal ganglia dysfunction, unilateral peripheral vestibular hypofunction, and motor disability are the significant clinical factors contributing to the severity and laterality of PS in patients with PD. The present findings improve our understanding on the pathogenesis of PS by unraveling an intimate link between the potential risk factors for the occurrence of PS and those related to the specific aspects of PS. Moreover, this study may facilitate the future longitudinal study investigating the predictors for more severe PS among patients with PD, by providing the clinical factors associated with the severity of PS. This approach may also help develop the novel rehabilitation or neuromodulation treatment for PS in patients with PD, such as the modulation of the erroneously perceived verticality.

## Data Availability Statement

The data that support the findings of this study are available on request from the corresponding author. The data are not publicly available due to privacy or ethical restrictions.

## Ethics Statement

The studies involving human participants were reviewed and approved by Institutional Review Board of Samsung Medical Center. The patients/participants provided their written informed consent to participate in this study.

## Author Contributions

YEH contributed to design and conceptualization of the study, drafting the manuscript, analysis and interpretation of data for intellectual content, and obtaining funding. D-WS contributed to the acquisition of data and analysis and interpretation of data for intellectual content and drafting and revising the manuscript. KK was involved in acquisition of data, analysis and interpretation of data for intellectual content. W-HC contributed to analysis and interpretation of data for intellectual content. SK was responsible for statistical analysis, analysis and interpretation of data for intellectual content. JWC contributed to design and conceptualization of the study, drafting and revising the manuscript, analysis and interpretation of data for intellectual content, and obtaining funding. All authors contributed to the article and approved the submitted version.

## Conflict of Interest

The authors declare that the research was conducted in the absence of any commercial or financial relationships that could be construed as a potential conflict of interest.

## Publisher’s Note

All claims expressed in this article are solely those of the authors and do not necessarily represent those of their affiliated organizations, or those of the publisher, the editors and the reviewers. Any product that may be evaluated in this article, or claim that may be made by its manufacturer, is not guaranteed or endorsed by the publisher.
