# Supplementary material for: Factors Contributing to the Severity and Laterality of Pisa Syndrome in Parkinson’s Disease
Source: Front Aging Neurosci. 2022 Jan 3;13:716990. doi: 10.3389/fnagi.2021.716990 (PMC8761952; doi:10.3389/fnagi.2021.716990)
Supplement: Supplementary file 1 [file Data_Sheet_1.docx]

**Supplementary table 1 Univariable analysis of clinical factors associated with PS tilting to the right**

|  | **PS tilting to the right**  **(n = 33)** | **PS tilting to the left**  **(n = 21)** | **OR** | **95% CI for OR** | ***p*** |
| --- | --- | --- | --- | --- | --- |
| **Age, years** | 65.8 ± 6.7 | 67.4 ± 6.0 | 0.96 | 0.88-1.05 | 0.392 |
| **Male, n (%)** | 20 (60.6) | 13 (61.9) | 0.95 | 0.30-2.90 | 0.924 |
| **Disease duration, years** | 7.5 ± 3.1 | 8.7 ± 3.8 | 0.90 | 0.76-1.05 | 0.187 |
| **Hoehn and Yahr stage** | 2.2 ± 0.4 | 2.2 ± 0.3 | 2.098 | 0.39-14.31 | 0.411 |
| **UPDRS-III** | 23.5 ± 6.0 | 21.7 ± 5.3 | 1.06 | 0.96-1.19 | 0.260 |
| **PD motor subtype, PIGD, n (%)** | 13 (39.4) | 10 (47.6) | 0.72 | 0.23-2.17 | 0.552 |
| **Asymmetry score of motor symptoms** | 5.6 ± 2.6 | 5.3 ± 2.7 | 1.04 | 0.84-1.29 | 0.706 |
| **Dominant side of motor symptoms, right, n (%)** | 16 (48.5) | 16 (76.2) | 0.29 | 0.08-0.95 | 0.048 |
| **Degree of SVV tilt** | 5.9 ± 3.3 | 6.1 ± 4.8 | 0.99 | 0.86-1.14 | 0.855 |
| **Direction of SVV, n (%)** |  |  |  |  |  |
| Right-sided SVV tilt | 19 (57.6) | 3 (14.3) | 11.88 | 2.97-62.61 | 0.002 |
| Normal SVV tilt | 6 (18.2) | 3 (14.3) | 3.75 | 0.77- 21.85 | 0.224 |
| Left-sided SVV tilt* | 8 (24.2)) | 15 (71.4) | NA | NA | NA |
| **Unilateral canal paresis, n (%)** | 4 (12.1) | 4 (19.0) | 0.59 | 0.12-2.77 | 0.488 |
| **Unilateral EMG hyperactivity**  **of paraspinal muscles, n (%)** | 24 (72.7) | 14 (66.7) | 1.33 | 0.40-4.39 | 0.635 |
| **Back pain, n (%)** | 15 (45.5) | 10 (47.6) | 0.92 | 0.30-2.77 | 0.876 |
| **BBS** | 49.6 ± 3.2 | 49.1 ± 2.9 | 1.05 | 0.87-1.26 | 0.608 |
| **LEDD, mg** | 562.6 ± 223.5 | 642.5 ± 204.7 | 0.10 | 0.10-1.00 | 0.193 |
| **Treatment regimen, n (%)** |  |  |  |  |  |
| Dopamine agonist | 3 (9.1) | 1 (4.8) | 0.67 | 0.02-10.86 | >0.999 |
| Levodopa + dopamine agonist | 26 (78.8) | 18 (85.7) | 0.48 | 0.02-4.11 | >0.999 |
| Levodopa * | 4 (12.1) | 2 (9.5) | NA | NA | NA |
| **BMI, kg/m^2^** | 23.8 ± 2.8 | 24.6 ± 2.4 | 0.89 | 0.70-1.10 | 0.298 |
| **MMSE** | 27.6 ± 2.1 | 28.5 ± 1.3 | 0.73 | 0.50-1.01 | 0.082 |
| **Education, years** | 11.8 ± 3.6 | 11.2 ± 3.6 | 1.05 | 0.90-1.23 | 0.512 |

*BBS, Berg Balance Scale; BMI, body mass index; LEDD, levodopa equivalent daily dose; MMSE, Mini-Mental State Examination; PIGD, postural instability and gait disturbance; SVV, subjective visual vertical; UPDRS-III, Unified Parkinson Disease Rating Scale motor score.*

*Data are given in mean±SD, unless otherwise noted.*

**reference*

**Supplementary table 2 Comparison of clinical factors between PD-PS patients with right- and left- sided SVV tilt**

|  | **Right-sided SVV tilt**  **(n = 32)** | **Left-sided SVV tilt**  **(n = 22)** | ***p*** |
| --- | --- | --- | --- |
| **Age, years** | 67.3 ± 5.1 | 66.1 ± 7.2 | 0.539 |
| **Male gender, n (%)** | 14 (63.6) | 13 (56.5) | 0.626 |
| **Disease duration, years** | 7.8 ± 2.6 | 7.9 ± 3.5 | 0.864 |
| **Hoehn and Yahr stage** | 2.3 ± 0.4 | 2.1 ± 0.3 | 0.151 |
| **UPDRS-III** | 23.2 ± 6.5 | 22.9 ± 5.5 | 0.882 |
| **PD motor subtype, PIGD, n (%)** | 9 (40.9) | 12 (52.2) | 0.449 |
| **PS tilting to the less affected side, n (%)** | 9 (40.9) | 17 (73.9) | 0.025 |
| **PS tilting to the right, n (%)** | 19 (86.4) | 8 (34.8) | < 0.001 |
| **Asymmetry score of motor symptoms** | 5.6 ± 2.5 | 5.2 ± 3.0 | 0.646 |
| **Dominant side of motor symptoms, right, n (%)** | 12 (54.5) | 16 (69.6) | 0.299 |
| **Degree of SVV tilt** | 7.9 ± 4.2 | 5.6 ± 2.9 | 0.016 |
| **Unilateral canal paresis, n (%)** | 1 (4.5) | 5 (21.7) | 0.090 |
| **Unilateral EMG hyperactivity**  **of paraspinal muscles, n (%)** | 20 (90.9) | 14 (60.9) | 0.019 |
| **Back pain, n (%)** | 8 (36.4) | 13 (56.5) | 0.175 |
| **BBS** | 48.7 ± 2.9 | 49.2 ± 2.7 | 0.568 |
| **LEDD, mg** | 540.7 ± 175.6 | 636.5 ± 229.0 | 0.211 |
| **Treatment regimen, n (%)** |  |  | 0.543 |
| Dopamine agonist | 2 (9.1) | 2 (9.7) |  |
| Levodopa + dopamine agonist | 17 (77.3) | 20 (87.0) |  |
| Levodopa | 3 (13.6) | 1 (4.3) |  |
| **BMI, kg/m^2^** | 24.4 ± 2.3 | 24.4 ± 2.3 | 0.937 |
| **MMSE** | 27.5 ± 2.3 | 28.0 ± 1.4 | 0.579 |
| **Education, years** | 12.0 ± 3.8 | 10.9 ± 3.8 | 0.317 |

*BBS, Berg Balance Scale; BMI, body mass index; LEDD, levodopa equivalent daily dose; MMSE, Mini-Mental State Examination; PIGD, postural instability and gait disturbance; SVV, subjective visual vertical; UPDRS-III, Unified Parkinson Disease Rating Scale motor score.*

*Data are given in mean±SD, unless otherwise noted.*

*Mann-Whitney or x2 test was used as appropriate.*
